# Cross‐seasonal legacy effects of arthropod community on plant fitness in perennial plants

**DOI:** 10.1111/1365-2745.13231

**Published:** 2019-07-08

**Authors:** Jeltje M. Stam, Martine Kos, Marcel Dicke, Erik H. Poelman

**Affiliations:** ^1^ Laboratory of Entomology Wageningen University Wageningen The Netherlands

**Keywords:** *Brassica oleracea*, community composition, community dynamics, herbivory, insect‐plant interactions, long‐term effects, seed set

## Abstract

1. In perennial plants, interactions with other community members during the vegetative growth phase may influence community assembly during subsequent reproductive years and may influence plant fitness. It is well‐known that plant responses to herbivory affect community assembly within a growing season, but whether plant–herbivore interactions result in legacy effects on community assembly across seasons has received little attention. Moreover, whether plant–herbivore interactions during the vegetative growing season are important in predicting plant fitness directly or indirectly through legacy effects is poorly understood.
2. Here, we tested whether plant–arthropod interactions in the vegetative growing season of perennial wild cabbage plants, *Brassica oleracea*, result in legacy effects in arthropod community assembly in the subsequent reproductive season and whether legacy effects have plant fitness consequences. We monitored the arthropod community on plants that had been induced with either aphids, caterpillars or no herbivores in a full‐factorial design across 2 years. We quantified the plant traits ‘height’, ‘number of leaves’ and ‘number of flowers’ to understand mechanisms that may mediate legacy effects. We measured seed production in the second year to evaluate plant fitness consequences of legacy effects.
3. Although we did not find community responses to the herbivory treatments, our data show that community composition in the first year leaves a legacy on community composition in a second year: predator community composition co‐varied across years. Structural equation modelling analyses indicated that herbivore communities in the vegetative year correlated with plant performance traits that may have caused a legacy effect on especially predator community assembly in the subsequent reproductive year. Interestingly, the legacy of the herbivore community in the vegetative year predicted plant fitness better than the herbivore community that directly interacted with plants in the reproductive year.
4. *Synthesis.* Thus, legacy effects of plant–herbivore interactions affect community assembly on perennial plants across growth seasons and these processes may affect plant reproductive success. We argue that plant–herbivore interactions in the vegetative phase as well as in the cross‐seasonal legacy effects caused by plant responses to arthropod herbivory may be important in perennial plant trait evolution such as ontogenetic variation in growth and defence strategies.

## 1 INTRODUCTION

Although the presence of species or interactions among species in ecological communities may be brief, the effect of their presence or their interaction may be traced back over a much longer time course after the initial species or interaction has passed. Such legacy effects can have important consequences for later species interactions and community dynamics (Kostenko, Voorde, Mulder, Putten, & Bezemer, 2012; Ohgushi, 2016; Utsumi, Ando, & Miki, 2010; Wurst & Ohgushi, 2015). Long after they have left, individual species may have prominent influences on community organization when they have a long‐term effect on the phenotype of a basal resource that structures communities (Wurst & Ohgushi, 2015). For example, root exudates from a plant can influence the soil microbiome and subsequent succession of plant communities (De Deyn, Raaijmakers, & van der Putten, 2004; van der Putten, 2003). Earthworms may alter soil nutrient composition and favour late successional plant species in establishing in plant communities (Mudrák & Frouz, 2018), whereas the soil biome may also stabilize legacies in vegetation composition caused by herbivore feeding (Egelkraut, Kardol, De Long, & Olofsson, 2018). The legacy of species interactions may be extensive when the effects cascade across trophic levels. The legacy of plant‐soil feedback may not only affect plant community composition, but also plant trait composition such as resistance to insect herbivores (Heinen, Sluijs, Biere, Harvey, & Bezemer, 2018) and the legacy of herbivory on plant community composition may affect the composition of insect and bird species over a period of decades (Nuttle, Yerger, Stoleson, & Ristau, 2011).

In plant–insect communities, legacies of species interactions are well‐characterized for species assemblies within a single season (Hernandez‐Cumplido, Glauser, & Benrey, 2016; Poelman, van Loon, van Dam, Vet, & Dicke, 2010; Wurst & Ohgushi, 2015; van Zandt & Agrawal, 2004). Presumably to reduce costs of defence in the absence of herbivores, many plant species only invest in enhanced levels of defence in response to actual herbivore attack (Karban, 2011; Mithöfer & Boland, 2012; Stam et al., 2014). These induced plant responses provide plants with enhanced resistance to the current attacker, but at the same time the induced plant phenotype potentially affects interactions with other community members, resulting in so‐called indirect plant‐mediated species interactions (Ohgushi, 2005; Utsumi et al., 2010). Because plant responses to herbivory are often specific for the guild or even herbivore species that attacks the plant (Bidart‐Bouzat & Kliebenstein, 2011; de Vos et al., 2005), each herbivore species may differentially affect other community members (Mathur et al., 2013; Rodriguez‐Saona, Chalmers, Raj, & Thaler, 2005; Stam et al., 2014). Therefore, each herbivore species may have unique effects on the assembly of the community within the growing season of that plant (Poelman et al., 2010; Stam, Dicke, & Poelman, 2018; Viswanathan, Narwani, & Thaler, 2005; van Zandt & Agrawal, 2004). These legacy effects by indirect plant‐mediated species interactions have been found to also affect reproductive fitness of annual plants (McArt, Halitschke, Salminen, & Thaler, 2013; Rusman, Lucas‐Barbosa, & Poelman, 2018) and may thus be important components of plant defence trait evolution (Poelman & Kessler, 2016).

In contrast with annuals, perennial plants have multiple growing seasons that typically consist of a distinct vegetative growing season followed by one or multiple years during which the plants flower and reproduce. When legacies of insect–plant interactions in the vegetative season extend across years into reproductive seasons, this may have important consequences for plant fitness (Ehrlén, 2002; West & Louda, 2018; Wurst & Ohgushi, 2015). Although induced responses to single herbivores may have season‐long legacies on plant‐associated insect community composition (Hernandez‐Cumplido et al., 2016; Poelman et al., 2010; Viswanathan et al., 2005; van Zandt & Agrawal, 2004), little is known about how long it takes for the community legacy of induced plant responses to decay, especially in the context of multiple growing seasons of perennial herbs (Gomez, van Dijk, & Stuefer, 2010; Karban, 2011; Underwood, 1998, 2012). Several examples show that herbivore‐induced plant responses may persist throughout several growing seasons in perennial trees (Haukioja, 1990; Haukioja, Suomela, & Neuvonen, 1985; Miller‐Pierce & Preisser, 2012; Nuttle et al., 2011; Young & Okello, 1998). This implies that legacy effects of plant–herbivore interactions can extend beyond a single growth season. Thereby, also in temperate regions where winters may cause plant‐associated communities to re‐assemble each year, long‐lasting legacies may connect assemblies across years (Karban, 2011; Wurst & Ohgushi, 2015). It is, thus, important to know whether such long‐lasting legacies of early herbivores also exist for the insect community associated with perennial herbaceous plants, especially when effects of community composition in a vegetative year affect plant–insect interactions and plant reproduction in subsequent years (Wurst & Ohgushi, 2015).

Here, we studied 2‐year legacy effects of early season herbivory on arthropod community composition and the consequences for fitness of an herbaceous perennial plant. In a field experiment over two consecutive years, wild perennial *Brassica oleracea* plants were inoculated early in the season with either of two specialist insect herbivore species from different feeding guilds (aphid or caterpillar), or no herbivore (control), in a full‐factorial design across years. Arthropod community composition was monitored in the vegetative and following first reproductive season, and at the end of the second year seed set was quantified. Specifically, we addressed the following questions: (a) Does early‐season herbivory by aphids or caterpillars affect arthropod community composition and plant fitness, either within or across years? (b) Does variation in arthropod community composition as a whole cause legacy effects on community composition and plant fitness across years? and (c) Which insect species and which plant performance traits are involved in the above processes? We discuss the data in the context of insect‐plant ecology and evolution of plant defences.

## 2 MATERIALS AND METHODS

### 2.1 Field site

Herbaceous wild perennial *B. oleracea* L. (Brassicaceae) plants, originating from Kimmeridge, Dorset, UK (50°36'N, 2°07'W) (Gols et al., 2008) were planted in a common garden in the vicinity of Wageningen University, The Netherlands. Seeds were sown in mid‐April 2012 and transplanted to peat soil cubes 11 days later. Seedlings were grown in a greenhouse until 4 w after sowing, after which they were placed outside to habituate them to field conditions. In week 21 (end of May) 2012, 72 plots of 12 plants each in a 4 × 4 square (omitting the central four plants to ensure equal plant neighbour effects) were established in the field (Stam et al., 2018). Within‐plot planting distance between plants was 1 m and plots were separated by a 4 m wide strip of a *Poa*/*Lolium* grass mixture. To ensure a uniform edge environment, two rows with plants of the annual *Brassica nigra* were planted at 4 m distance from the plots at the border of the field, 1 m between rows and 0.5 m between plants within rows. The seeds for these plants had been collected from wild *B. nigra* plants in the vicinity of Wageningen, the Netherlands, and were sown and treated similar to the *B. oleracea* plants as described above. Plots and edge were regularly manually weeded and grass strips were regularly mown. The plants were experimentally infested with herbivores early in the season of two subsequent years (2012 and 2013, see below) and exposed to naturally occurring arthropods during the rest of season. In the winter period (January 8–April 3 2013), plants were protected from severe freezing/dehydrating conditions by covering the whole *B. oleracea* field with a cloth (26 g/m^2^, AMEVO, the Netherlands). The *B. nigra* plants for the border were re‐sown and planted next spring, similar as described above (planting in the field in week 21, end of May 2013).

### 2.2 Legacy effects: Two‐year common garden experiment

In order to study legacy effects of herbivore induction on community composition within and across seasons, as well as legacy effects of variation in the community as a whole across seasons, we manipulated the first herbivores arriving on the individual *B. oleracea* plants. In week 22 (end of May) of 2012, six days after the seedlings were planted, we infested all plants of the 72 plots with either five adult aphids (*Brevicoryne brassicae* L. (Hemiptera: Aphididae)) (A), three caterpillars in 2nd larval stage (*Plutella xylostella* L. (Lepidoptera: Yponomeutidae)) (C), or left plants uninfested (N). The insects were obtained from the stock rearing of Wageningen University, where they were reared on Brussels sprouts plants (*B. oleracea* var. *gemmifera* cv Cyrus) under greenhouse conditions (21 ± 1°C, 50%–70% relative humidity, 16L: 8D cycle). The two herbivore species are among the most common herbivores on *B. oleracea* and we selected an aphid and caterpillar species because they are known to induce widely different plant responses (Stam et al., 2018). All plants in a plot were infested with the same treatment by carefully introducing herbivores using a fine brush, yielding 24 plots for each herbivore treatment in 2012 (Table 1). In week 20 (mid May) of the second growth season (2013), the 24 plots that had received the same herbivore treatment in 2012 were assigned to three groups of eight plots, each receiving one of the three herbivore treatments (A, C, N). This resulted in a full factorial design of induction combinations across the two seasons and a total of nine unique treatments each replicated with eight plots (Table 1).

**Table 1:** Early‐season herbivory treatments in two consecutive years, applied in a common‐garden field experiment on wild perennial *Brassica oleracea* plants. ‘**M**’ indicates treatment plots that were monitored in both 2012 and 2013; the other plots were monitored in 2013 only

Early‐season herbivore year 1 (2012)	Early‐season herbivore year 2 (2013)	Abbreviation
Aphids *Brevicoryne brassicae*	Aphids *B. brassicae*	AA
Aphids *B. brassicae*	Caterpillars *Plutella xylostella*	AC
Aphids *B. brassicae*, **M**	No early‐season herbivore	AN
Caterpillars *P. xylostella*	Aphids *B. brassicae*	CA
Caterpillars *P. xylostella*	Caterpillars *P. xylostella*	CC
Caterpillars *P. xylostella*, **M**	No early‐season herbivore	CN
No early‐season herbivore	Aphids *B. brassicae*	NA
No early‐season herbivore	Caterpillars *P. xylostella*	NC
No early‐season herbivore, **M**	No early‐season herbivore	NN

In 2012, we monitored eight plots of each of the three herbivore treatments (A, C, N). We had to restrict the number of observations in this year to allow for intensive monitoring of the within‐year effects of early season herbivory. We assumed that the subset of eight replicate plots within a treatment was representative for the full 24 replicates of early‐season herbivory treatments that were prepared in the first year to allow for the full factorial design of treatment interactions across years. In 2012, we collected data over 12 time points during the season on a weekly basis by repeatedly monitoring the same four plants of a plot for their arthropod community.

In 2013, the 72 plots representing all nine treatments were monitored to focus on across‐year effects of individual herbivore treatments as well as legacy effects of community composition across years. We selected four plants per plot that showed flower buds in week 20 (non‐flowering plants were excluded from monitoring) for monitoring of the arthropod community. Because a larger number of plots and thus plants was monitored in 2013 and plants were much larger than in 2012, the community on these plants could be monitored two times: during early season (week 21–25) and mid/late season (week 25–33). Within each of the two time periods, plots with different treatments were monitored in a random order to minimize time‐effects on in treatment comparisons. Since we covered the same weeks of monitoring in both years, we could asses presence and abundance of herbivores and predators across the full season for each treatment in both years despite the necessary compromise on sampling intensity in 2013.

Monitoring plants for arthropods in both years occurred through visually screening for all life stages of all living insects and other invertebrates such as spiders and slugs (because the community is arthropod dominated it is here collectively referred to as ‘arthropod community’) on the upper‐ and lower parts of each leaf and flower. Both herbivores and carnivores (predators plus parasitoids) were recorded. However, fast‐flying insects, such as adult parasitoids, butterflies and pollinators were not recorded as their presence could not be accurately assigned to a single plant. Parasitized aphids (‘mummies’) were identified as aphid parasitoids; parasitoids of caterpillars were identified by their cocoons once emerged from their caterpillar host in the field. The number of individuals per species was recorded per plant, taking all life stages together. See Table S1 for a list of observed arthropod species. To test which plant performance parameters corresponded with insect community assembly, we also recorded plant total height and number of leaves in both years at the same moments when plants were monitored for arthropods (12 times in 2012 and two times in 2013). In addition, in the reproductive season (2013) the number of flower racemes (unbranched stalks bearing flowers) was recorded. During the winter period between the 2 years (October 2012–March 2013), a few randomly selected plants in the field were screened for the presence of arthropods, but none were found.

### 2.3 Seed harvest

To test whether either early‐season herbivory treatments or total arthropod community composition exerted legacy effects on plant fitness, seeds of all monitored plants were harvested. Seeds formed during the first plant reproductive season (2013) were collected after the second period of monitoring, from week 34 to the beginning of week 39. Racemes with dry seed pods were cut and placed in a paper bag per plant. A cloth underneath the plant collected seeds falling during the harvesting process. Seeds were separated from remaining plant material and the full seed yield per plant was weighed. The number of seeds per plant was computed by dividing the total weight of the seed batch by the weight of 100 seeds of the same plant, and multiplied by 100.

### 2.4 Statistical analyses

#### 2.4.1 Early‐season herbivory effects on community composition

We first tested the effects of early‐season herbivory treatments (aphids, caterpillars or none) on the herbivore or carnivore community composition within and across years on plot level, averaging the abundance of each species over the four plants per plot. For the first year (2012), abundance of each species was cumulated over all 12 monitored time points to obtain the community composition over the whole season for each plot. Only the 24 plots that were monitored that year were used for this (‘M’, Table 1). For the second year (2013), abundance of each species was similarly cumulated over the two time periods, and all 72 plots were used (Table 1), except for plots in which all plants had died over winter (*n* = 4). Redundancy analyses (RDAs) were used to test the effects of the early‐season herbivory treatment applied to a plot (aphid, caterpillar or none) in the first or second year, on the community composition per plot (cumulated species abundance) in the first or second year. Tests were performed with a Monte Carlo permutation test with 499 unrestricted permutations. A linear method was assumed valid as the length of species data gradient was <3 turnover (*SD*) units long (Šmilauer & Lepš, 2014). Species numbers were log (*y* + 0.25) transformed prior to analyses to reach best model fit. In these analyses, abundances of *P. xylostella* and *B. brassicae* were made supplementary (excluding them from ordination analysis, but projecting them afterwards in biplots) to exclude effects of herbivores that were directly manipulated by our treatments.

#### 2.4.2 Early‐season herbivory effects on plant fitness

First, to test the plant fitness effects of the early‐season herbivory treatments applied in the first and second as well as the interaction between both years, the number of seeds per individual plant were analysed by two‐way ANOVA. Second, to assess in more detail which combinations of induction treatments in the first and second year specifically affected plant fitness, we conducted a one‐way ANOVA on all nine treatment combinations (Table 1). Third, we grouped treatments that had the same herbivore inoculation (aphid, caterpillar or none) in the first year, or the same herbivore inoculation in the second year to test whether first or last herbivore treatments were more predictive for plant fitness (Table 1). On each of these six groups we conducted a one‐way ANOVA, followed by an LSD post‐hoc test if effects were significant. For all tests, seed set of individual plants from all monitored plots in 2012 and 2013 were used (Table 1), except for plants that had died before they produced seeds (*n* = 52 of 288 monitored plants). Number of seeds per plant was double square‐root transformed to meet assumptions of normal distribution and homogeneity. ANOVA tests were carried out in SPSS Statistics for Windows, Version 22.0.0.1 (Armonk, NY, USA; IBM corp.).

#### 2.4.3 Community legacy effects within and across years: Structural equation model

To relate early season herbivory treatments, herbivore and carnivore community composition in either year to seed set of the perennial plants, we used structural equation modelling (SEM). SEM was used to address two questions: (a) whether early season herbivory treatments affected community ordination and plant fitness within and across two seasons, and (b) whether variation in the composition of the herbivore and carnivore community affected community composition and plant fitness within and across years.

To obtain one value per plot for each of the variables, arthropod community composition was represented by ordination scores of field plots on the first axis of a Principal Component Analysis (PCA; see Supporting Information for additional note). For PCAs, herbivore and carnivore community data in 2012 and 2013 were similarly prepared as described above for RDAs, except that for all variables, only the 24 plots were used that were monitored for community composition in both 2012 and 2013 (‘M’ in Table 1). Two plots of which all plants had died in 2013 were excluded. Species data were log (*y* + 0.25) transformed prior to PCA, with abundance of *P. xylostella* and *B. brassicae* made supplementary. The resulting ordination score on the first principal component axis of each field plot was used as input for the SEM. Herbivory treatments per year were included as either aphid (A), caterpillar (C), or none (N) (Table 1). Average seed set per plot for SEM were square‐root transformed prior to analysis to meet assumptions of normality of SEM. The model best fitting the data was selected by removing non‐significant paths from the model. In SEM, the goodness of fit of the model is assessed by comparing the observed and model‐predicted covariances with a *χ* ^2^ test. The model is acceptable (there is reasonable fit between model and the data) when the *χ* ^2^ values have an associated *P* > 0.05 (Grace, 2006). SEM analyses were carried out with ‘sem’ package in r (version 3.0.1, R Development Core Team 2013).

#### 2.4.4 Ordination of species involved in legacy effects: Principal component analyses

Species ordination plots were made to obtain more detailed information on which individual species were involved in legacy effects on community composition and plant fitness. For the two most interesting paths of the SEM described (see Section 3), scatterplots were made indicating which species likely occur in the same plot (e.g. long species‐arrows pointing in the same direction). First, to show the relationship of carnivore species occurrence in plots in the first and second year, two scatterplots of carnivore species in either year were made by PCA as described above, using the same data as was used for SEM input (for these original scatterplots, see Supplementary material). The two scatterplots were then overlaid, such that the carnivore species ordination of both years was depicted in one image (See Supporting Information for justification of this method).

Second, to show the relationship of the herbivore species present in the first year with seed set of those plants in the following year, another PCA biplot was made depicting ordination of herbivore species in the vegetative season (2012) and seed set in the reproductive season (2013). Seed set in 2013 was a supplementary variable that did not influence species ordination. Afterwards an arrow indicating the direction of plots with increasing seed set was projected onto the herbivore species scatterplot. (The RDA biplots shown in the Supporting Information for herbivore community 2012 + seed set; carnivore community 2012 + seed set; and carnivore community 2013 + seed set were similarly obtained.)

All ordination analyses (RDA, PCA) were executed with Canoco 5.04 for Windows (ter Braak & Šmilauer, 2012). See Supporting Information for more details on interpretation of ordination plots using the biplot rule.

#### 2.4.5 Plant performance traits involved in herbivore‐community legacy effect on plant fitness: Structural equation model

We performed another SEM to investigate whether and how plant performance traits mediated first‐year herbivore community legacy effects on seed set in the second, reproductive year. Herbivore community in 2012 was represented by PCA scores similar to those in the first SEM. We used the plant traits ‘height’ and ‘number of leaves’ in both years as indicators of plant size, and ‘number of flower racemes’ in 2013 as indicator of the amount of reproductive tissue. For plant traits in 2012, measurements during the peak of the arthropod season (week 35; Stam et al., 2018) were taken as input. For plant traits in 2013 the situation was different due to a reduction of number of leaves while the plants started flowering and subsequently formed seeds as the season progressed. Therefore, in 2013 measurements when plant traits were on average at their maximum value were taken as input: early season (week 21–25) for number of leaves; mid/late season (week 25–33) for plant height and number of flower racemes. Number of leaves in 2013 was square‐root transformed to obtain normality. Similar as for the first SEM on community legacy effects, average seed set per plot in 2013 was used and data were square‐root transformed for normality.

## 3 RESULTS

### 3.1 Early‐season herbivory effects on community composition and plant fitness

**Figure 1:**
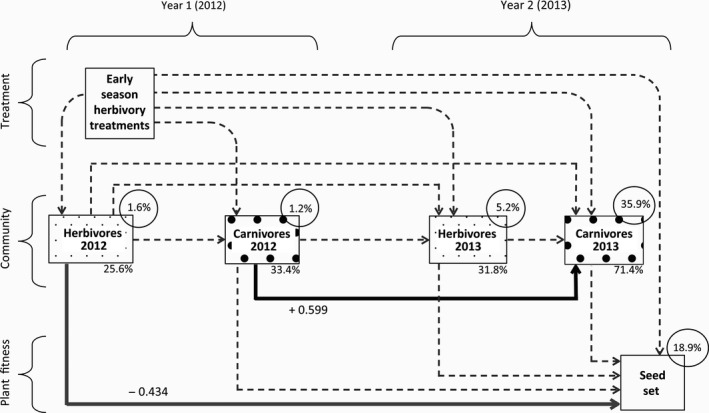
Structural equation model (SEM) of relations between early‐season herbivory treatments in the first year (2012), herbivore and carnivore community composition in the first (2012) and second year (2013) and seed set of plants in the second year (2013). For community composition, scores on the first ordination axis of principal component analysis (PCA) were used, of which the % explained variation is mentioned below each block. *R* ^2^ values (%) as shown on top of each endogenous explanatory variable (circle) give the explained variation by all paths to that variable by the SEM. Dotted lines indicate non‐significant effects, while continuous lines show significant effects, with their standardized path coefficients: black line for a positive relationship, grey line for a negative relationship

Early‐season herbivory treatments in the first and second season had no effect on herbivore or carnivore community composition either within the year or in the following year, although early season herbivory in the first year had a near‐significant effect on the carnivore community composition in the following year (*p* = 0.056, Table 2). Also, in the SEM on community legacy effects (see below, Figure 1), none of the paths significantly related early‐season herbivory treatments in the first year to herbivore or carnivore community in either year, nor to plant seed set.

**Table 2:** Results of Redundancy Analysis (RDA) of the early season herbivory treatments (aphid, caterpillar or no herbivory) in either year on wild perennial *Brassica oleracea* plants, to the herbivore and carnivore community composition within and across years. Treatments applied early in the season in 2012 and 2013 were either *Brevicoryne brassicae* aphids, *Plutella xylostella* caterpillars or no herbivory. Percentages show the % cumulative explained variation by the first two RDA axes; *F*‐values are pseudo‐*F* values of Monte Carlo Permutation test with 499 unrestricted permutations. Degrees of freedom of the explanatory variable ‘year of herbivory’ was in all cases 2; *α* = 0.05

Year early herbivory	Affected community	%	*F*	*p*
2012	Herbivores 2012	6.82	0.8	0.758
2012	Carnivores 2012	8.94	1.0	0.422
2012	Herbivores 2013	2.00	0.7	0.814
2012	Carnivores 2013	6.02	2.1	0.056
2013	Herbivores 2013	3.29	1.1	0.374
2013	Carnivores 2013	1.40	0.5	0.828

**Figure 2:**
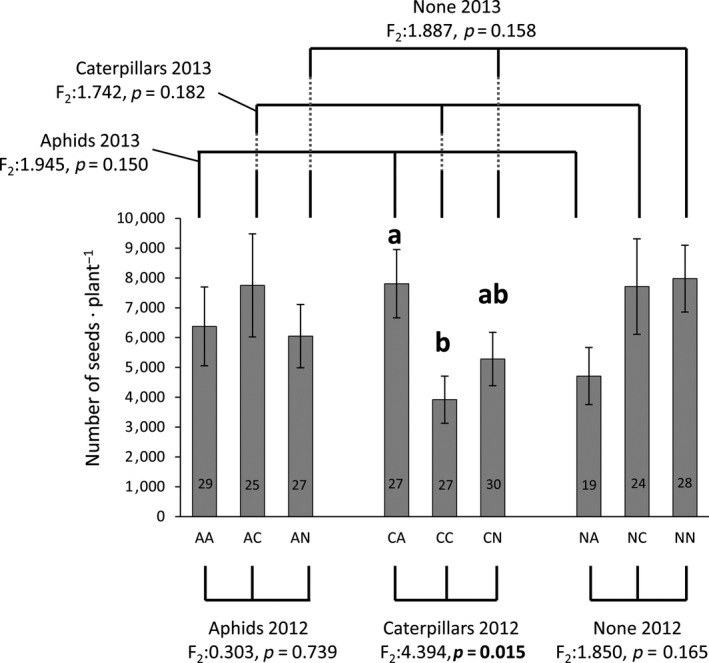
Average seed set (±*SE*) per plant in 2013 of wild perennial *Brassica oleracea* plants was affected by sequential early season herbivore treatments in two consecutive years. Abbreviations for early‐season herbivore inoculation: A: aphids *Brevicoryne brassicae*, C: caterpillars *Plutella xylostella*, N: no herbivores (None); the first letter indicates inoculation in the first year (2012) and the second letter inoculation in the second year (2013). As the interaction between herbivory treatments in 2012 * herbivory treatments in 2013 was significant, additional ANOVA tests were performed to gain insight which sequence of early herbivory in 2012–2013 affected seed set. Treatments were grouped to have the same herbivore inoculation in the first year (2012), or the same herbivore inoculation in the second year (2013), but that differed in herbivore species in the following or previous year, respectively. ANOVA results for each group are shown; **bold** *p*‐value indicates a significant difference in number of seeds between treatments tested (*α* = 0.05). Different letters above the bars indicate significant different numbers of seeds between treatments in that group. Number of replicates per treatment are shown within each bar

Although seed set of individual plants was not affected by the induction treatment of early‐season aphid, caterpillar or no‐herbivore feeding in either the first, vegetative year (ANOVA: *F*_2_:0.317, *p* = 0.729), or in the following, reproductive year (ANOVA: *F*_2_:1.068, *p* = 0.345), the interaction between herbivore treatments applied in the 2 years did significantly affect seed set (ANOVA: *F*_4_:2.600, *p* = 0.037). Only when in the first year caterpillars had been placed on the plants, seed set of individual plants differed between treatments applied in the second year: plants sequentially induced with caterpillars in both years (CC) produced fewer seeds than plants that had received caterpillars in the first year and aphids in the next year (CA); while seed set of plants infested with caterpillars in year 1 followed by no early‐season herbivory treatment in year 2 was intermediate (CN) (Figure 2). Plant seed production was thus determined by an interaction of our treatment of early herbivore presence in the vegetative year and following first year of flowering, rather than determined by the early herbivore attack in the flowering year alone. It is important to note that after the induction treatments plants were exposed to natural arthropod colonization and thus plant fitness measured is an effect of the treatments interacting with two years of biotic and abiotic events.

### 3.2 Legacy of whole arthropod community across years

Although our early‐season herbivory treatments did not affect community composition in the same or next year (above), herbivore and carnivore community composition in both the vegetative year (2012) and first flowering year (2013) did reveal legacy effects of total communities across years. A SEM that included herbivore and carnivore community composition provided a good fit to the data, and showed that especially the carnivore community was shaped by legacy effects (whole‐model fit: $X_{10}^{2}$ = 6.531, *p* = 0.769). Carnivore composition in the first year had a significant effect on the carnivore composition in the following year (SEM, *z*: 3.43, *p* < 0.001, Figure 1). This was not mediated by herbivore community within or across years, as none of those paths were significant (Figure 1). Also, the near‐significant effect of early‐season herbivory to carnivore community composition across years (Table 2) was not seen back in this SEM (Figure 1).

Second, herbivore community composition in the plants’ vegetative season (2012) correlated with seed set in the following, reproductive season of the plants (2013) (SEM, *z*: −2.21, *p* = 0.027). Also this was not mediated by either herbivore or carnivore community composition within or across years, as none of the intermediate paths were significant (Figure 1). Thus, mainly across‐year effects, rather than within‐year effects, influenced carnivore community composition and plant fitness.

### 3.3 Arthropod species involved in community legacy effects

**Figure 3:**
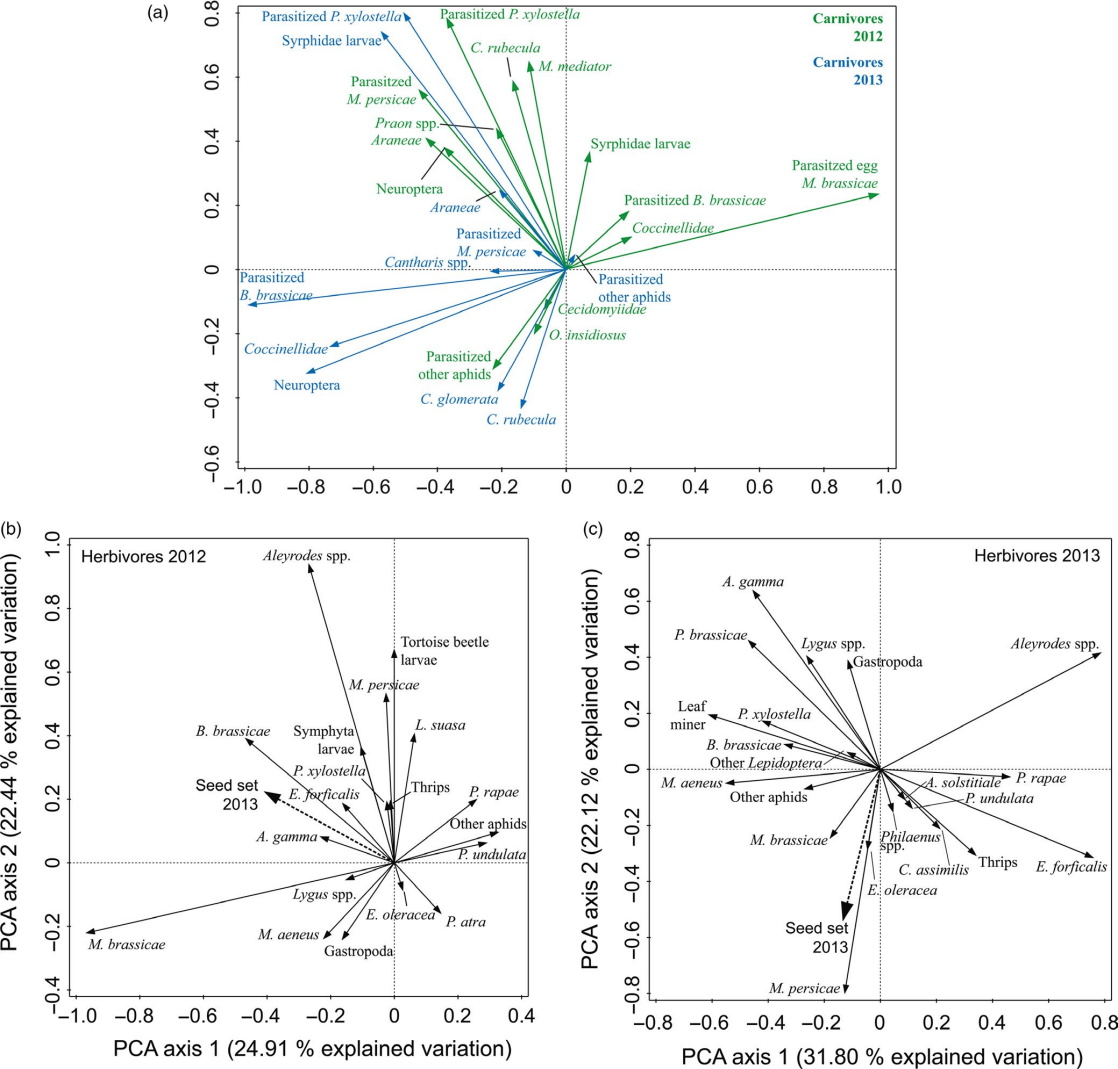
Principal component analysis (PCA) biplots showing species ordinations of (a) carnivore community in 2012 and 2013 (one combined overlay plot), (b) biplot of herbivore community in 2012 with variable ‘Seeds’ (seed set 2013), and (c) biplot of herbivore community in 2013 with variable ‘Seeds’ (seed set 2013). Input variables for PCA were the same as used for SEM. Long arrows pointing in the same direction show a positive correlation, whereas long arrows pointing in opposite directions show a negative correlation. Short arrows indicate a weak effect (low amount of explained variation). For a list of species, see Table S1 [Colour figure can be viewed at wileyonlinelibrary.com]

Especially parasitoids were involved in the cross seasonal correlation between the first‐year carnivore community to the second‐year carnivore community (Figure 3a, Table S1 and Figure S1). For example, parasitoids associated with the caterpillar *P. xylostella* occurred in high abundances on the same plots in both years (e.g. long arrows of the same species in either year pointing in the same direction in the PCA plot). The other way around, parasitoids of, for example, the aphid *B. brassicae* or the parasitoid *Cotesia rubecula* that is parasitizing *Pieris* caterpillars were abundant in one year, but low in abundance in the second year (or vice versa, e.g. long arrows pointing in opposite directions in the PCA plot). The abundances of predators belonging to, for example, the Neuroptera or Syrphidae larvae, however, did not show a strong correlation across years (e.g. arrows almost perpendicular to each other; Figure 3a). Spiders (Araneae) and ladybeetles (Coccinellidae) showed positive or negative relations across years, respectively, although for these species effect sizes were small.

Interestingly, the abundance of some specific herbivore species that were present in plots in the vegetative year corresponded with plant fitness a year later (Figure 3b), whereas no strong negative correlations between herbivores in 2013 and seed set were found (Figure 3c). Abundance of two flea beetle species (*Phyllotreta undulata* and *Pachygaster atra*) in the vegetative year corresponded with reduced seed set of plants. Plants that were less frequently attacked by these beetle species had higher numbers of other herbivore species such as the generalist caterpillar *Mamestra brassicae*, whiteflies (*Aleyrodes* spp.), and the specialist aphid *B. brassicae* (Figure 3b). Thus, mainly herbivore species present in the first vegetative growth season correlated with plant seed production in the second year when plants flowered for the first time, and these effects were more pronounced than the direct effect of herbivores colonizing plants in the first flowering year.

### 3.4 Community legacy to plant fitness mediated by plant performance traits

**Figure 4:**
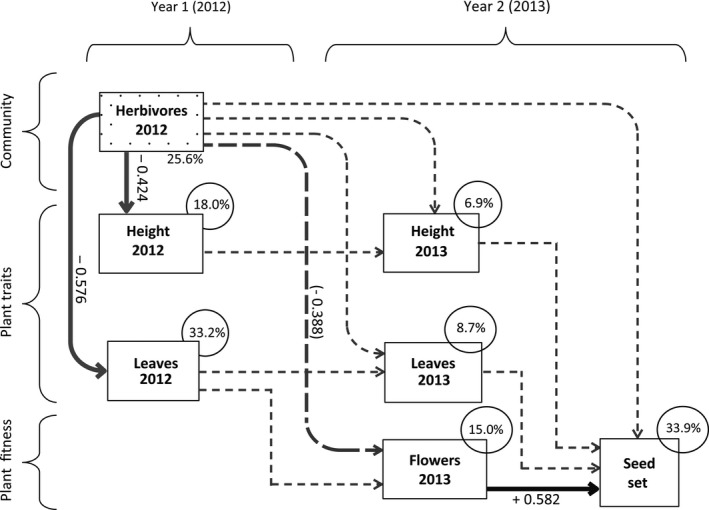
Structural equation model (SEM) of relations between herbivore community composition in the first year (2012), plant traits in the first, vegetative year (2012) (maximum height and number of leaves) and second, reproductive year (2013) (maximum height, number of leaves and number of flower racemes), and seed set of plants in the second, reproductive year (2013). For herbivore community composition, scores on the first ordination axis of principal component analysis (PCA) were used, of which the % explained variation is mentioned below the block of herbivores 2012. *R* ^2^ values (%) as shown on top of each endogenous explanatory variable (circle) give the explained variation by all paths to that variable by the SEM. Dotted lines indicate non‐significant effects, while continuous lines show significant effects, with their standardized path coefficients: black line for a positive relationship, grey line for a negative relationship. The path from herbivore community in 2012 to the number of flower racemes in 2013 was a near‐significant (*p* = 0.054) negative relationship

Plant traits such as height and number of leaves were found to mediate the effects of the herbivore community in the vegetative season to plant fitness in the following reproductive season (Figure 4). The SEM had good data fit ($X_{15}^{2}$ = 21.988, *p* = 0.108) and revealed that herbivore community composition in the first year significantly correlated with plant height (SEM, *z*: −2.14, *p* = 0.032) and number of leaves (SEM, *z*: −3.23, *p* = 0.001) within the first year (2012). A near‐significant path was found for the first‐year herbivore community correlating with the number of flower racemes in the next year (SEM, *z*: −1.93, *p* = 0.054). Subsequently, the number of flower racemes in the second season was positively correlated with the number of seeds in the first reproductive season (SEM, *z*: 3.28, *p* = 0.001). However, plant performance traits (height, number of leaves) did not correlate with each other across the two years. In conclusion, herbivore community composition in 2012 affected seed set in 2013 indirectly through plant performance traits, although the connecting chain of mediating plant traits via flower racemes was just not significant.

## 4 DISCUSSION

Despite the absence of an early season herbivore effect on community assembly, our study indicates that insect community assembly on perennial plants is affected by cross‐seasonal legacy effects of community composition across years. The carnivore community in the first vegetative growing season correlated with carnivore community assembly after winter in the second year when plants flowered for the first time. Moreover, the identity of early‐season herbivores colonizing plants in both years interacted in an effect on plant fitness. We identified that especially the herbivore community in the vegetative year predicted plant fitness rather than the community that directly interacted with the plant in the reproductive season. Our data indicate that the mechanism behind this legacy effect on plant fitness lies in the effect of herbivores on plant traits. The herbivore community in the first year corresponded with plant performance traits such as leaf number and plant size that determine capacity to flower in the subsequent year. Here, we discuss the role of plant traits in mediating legacy effects of the arthropod community as well as the consequences for plant trait evolution in perennial plants.

### 4.1 Plant phenotypic plasticity to herbivory

Plant responses to herbivore feeding have been increasingly recognized to mediate community assembly within a growth season (Hernandez‐Cumplido et al., 2016; Poelman et al., 2010; Stam et al., 2018; Viswanathan et al., 2005; van Zandt & Agrawal, 2004). In our earlier work, we identified that the herbivore species introduced as early‐season herbivores on the seedlings in 2012 differentially affected arthropod community assembly within the vegetative growing season (Stam et al., 2018). Moreover, order of herbivore arrival further modulated community assembly within this vegetative season, resulting in variation in community composition at the end of the first season (Stam et al., 2018). The absence of effects in the current study may be due to the use of a smaller sample size, analysis of insect communities as average over a season and not in time series of repeated measurements as in our earlier work (Stam et al., 2018), or context dependency of priority effects depending on yearly variation in community context. Despite the absence of a treatment effect by early‐season herbivory on community assembly in our current study, our SEM analyses as well as the plant fitness analyses for herbivore treatments indicate that herbivore‐plant interactions play a role in cross‐seasonal legacies of arthropod community assembly on perennial plants. The composition of the predator community in 2012 predicted the predator community on plants in 2013, and these were largely determined by specialist parasitoids that are strongly connected to specific herbivore hosts. Moreover, SEM analyses revealed that the herbivore community in the vegetative season correlated with plant traits such as height and number of leaves that carried over to the next season, potentially mediating the legacy on community composition in the next reproductive season.

Herbivores in the first year affected the plant phenotype such as its biomass in the next year, and were marginally significantly predictive for the parasitoid community in the next year. This indicates that the legacy of plant‐herbivore interactions cascades across trophic levels by affecting parasitoid communities in a subsequent year (Hernandez‐Cumplido et al., 2016). Plant biomass (in terms of height and number of leaves) is well‐known to positively correlate with herbivore presence and to cascade on increased predator abundance (Begon, Howarth, & Townsend, 2014; Schmitz, Krivan, & Ovadia, 2004). However, since our SEM could not connect the full path of legacy effects of plant–herbivore interactions to predator communities in subsequent years and our treatments of early‐season herbivory did not significantly affect community composition, it remains speculative what caused the co‐variation of predator communities over years. For parasitoids, density dependent processes of higher parasitoid abundance by increase in host presence is likely to have contributed to cross seasonal legacy effects in predator communities (Bezemer & Mills, 2001; Kos et al., 2011; Waage, 1983). Alternatively, parasitoid abundance in our study was not herbivore‐density‐dependent (Heimpel & Casas, 2008), and for example caused by genotypic variation in plant traits such as herbivore‐induced plant volatiles that are used in host location (Poelman, Oduor, et al., 2009a). Although individual plants were randomly planted in plots, we cannot exclude that plots differed in genotypic makeup and traits that affect parasitoid abundance (Lannér‐Herrera, Gustafeson, Filt, & Bryngelsson, 1996). Moreover, the connection between predator communities across years may include effects by predator interactions such as intraguild predation or non‐consumptive interactions (Frago & Godfray, 2014). Nevertheless, our data hint that legacy processes known from other study fields such as plant–soil feedback (Heinen et al., 2018; Kostenko et al., 2012; Mudrák & Frouz, 2018), herbivore–plant community interactions (Dong et al., 2018) or legacies of land use (Cusser, Neff, & Jha, 2018; Hahn & Orrock, 2015), may also contribute to cross‐seasonal community assembly on perennial plants.

### 4.2 Fitness of perennial plants

Both our ANOVA analyses of plant fitness as well as SEM analyses revealed that presence of herbivores in the first and vegetative year is most predictive for plant fitness in the subsequent reproductive year. Herbivores in the vegetative year affected plant biomass (height and number of leaves) that corresponded with decreased production of flowers and seed production in the second year. We found that specific herbivore species in 2012 corresponded with decreased seed production in 2013, that is, flea beetles. The beetles are known to particularly colonize *B. oleracea* early in its growth and respond to induction of plants by caterpillar feeding (Poelman, van Loon, van Dam, Vet, & Dicke, 2009b; Poelman et al., 2010). Plant responses to caterpillar feeding increase beetle abundance on caterpillar‐induced plants (Poelman et al., 2010). This may explain the lowest seed production of plants that received caterpillar induction in both years and the correlation of beetle abundance in 2012 with plant fitness. Our data cannot provide causal support for the hypothesis and it remains to be identified if flea beetles are indeed among the most important agents that reduce fitness of perennial *B. oleracea* when particularly feeding on plants in the vegetative year. For perennial plants it has been debated whether events in the vegetative years before reproduction or events during reproductive years are most predictive for plant fitness (Boege & Marquis, 2005). Our data match emerging consensus that events during the vegetative year profoundly influence plant fitness in subsequent years (Boege & Marquis, 2005; Ehrlén, 2002). Herbivore feeding in the vegetative year may cause reduction in photosynthetic capacity and allocation of resources from growth into defence, resulting into effects on plant biomass and defence phenotype (reduction in plant height and number of leaves in our study). The biomass loss and investment in defence to herbivores, carries over to subsequent years in which plants have reduced capacity to form reproductive tissues. The cumulative herbivore load in both vegetative and flowering years has been identified to reduce growth compensation for flower damage in a perennial thistle (West & Louda, 2018). Thus, plant‐animal interactions during the vegetative year have lifetime consequences for perennial herbs (Ehrlén, 2002). These effects may be strengthened by legacy effects of plant–animal interactions in the vegetative year to plant‐animal interactions in reproductive years. This includes the likelihood of herbivore attack (McArt et al., 2013; Poelman et al., 2010; van Zandt & Agrawal, 2004), predator presence (Hernandez‐Cumplido et al., 2016; Utsumi et al., 2010) as well as interactions with pollinators (Kessler & Halitschke, 2009). Plant responses to herbivory in terms of growth form, defence and flower traits have been identified to affect pollinator visitation and result in effects on seed production (Rusman et al., 2018).

### 4.3 Future perspective

The importance of herbivory in vegetative years for plant fitness as well as plant–animal legacy effects on community assembly across seasons suggests that these effects are also reflected in growth‐defence strategies of perennial plants. Costs of defence in vegetative years as well as legacies of plant‐mediated species interactions may prove to be explanatory factors in the evolution of plant ontogenetic trajectories of growth‐defence trade‐offs (Boege & Marquis, 2005). In this research, an important challenge is to identify whether communities as a whole or specific species drive plant‐mediated interactions and its fitness consequences (Guimarães et al., 2017; Poelman, 2015; Poelman & Kessler, 2016; Ohgushi, 2016). Investigating this is important for our understanding of the evolution of plant–insect communities.

## AUTHORS' CONTRIBUTIONS

J.M.S., M.D. and E.H.P. conceived the ideas and designed methodology; J.M.S. collected the data; J.M.S. and M.K. analysed the data; E.H.P. and J.M.S. led the writing of the manuscript. All authors contributed critically to the drafts and gave final approval for publication.

## Data Availability

Data deposited in the Dryad Digital Repository: https://doi.org/10.5061/dryad.g8k7261 (Stam, Kos, Dicke, & Poelman, 2019).
