# Supplementary material for: Cross‐seasonal legacy effects of arthropod community on plant fitness in perennial plants
Source: J Ecol. 2019 Jul 8;107(5):2451–63. doi: 10.1111/1365-2745.13231 (PMC6774310; doi:10.1111/1365-2745.13231)
Supplement: Supplementary file 1 [file JEC-107-2451-s001.pdf]

Supplementary Information belonging to:

Cross-seasonal legacy effects of arthropod community on plant fitness in perennial plants

Jeltje M. Stam[1]; Martine Kos[1]; Marcel Dicke[1] and Erik H. Poelman[1*]

[1]Laboratory of Entomology, Wageningen University, P.O. Box 16, 6700 AA Wageningen, the Netherlands

*Corresponding author: erik.poelman@wur.nl

Supplementary material

Interpretation of ordination plots using the biplot rule

The ordination analyses of community compositions in either year resulted in PCA biplots. These graphs show on the first two ordination axes the ordination of response variables, in this case the arthropod species in the community, and explanatory variables, in this case plant seed set. Both are depicted with an arrow from the origin to their ordination on (axis 1, axis 2). PCA graphs can be interpreted using the biplot rule (Šmilauer and Lepš, 2014), in which the position of the arrow-tip representing a species is projected with right angles to the (imaginary) line extending through the arrow representing plant seed set. Species whose arrows are long and point in the same direction as the arrow representing seed set are likely to occur on plants that have a large seed set. Contrasting species with an arrow in the opposite direction of the explanatory variable are more likely to occur on plants that have a small seed set. Finally, if the response and explanatory arrow are perpendicular to each other, then they show no correlation. Furthermore, the shorter the arrow is, the lower the explained variation.

The interpretation between two or more response variables (e.g. between species) is similar; so species whose arrows are long and point in the same direction are likely to occur together on the same plant.

From Principal Component Analysis to Structural Equation Model

The percentage of explained variation by the first PCA axis of herbivore and carnivore community in the first year and second year (Figures 3b,c and Supplementary Figures 1a,b,c) does not exactly correspond with the percentages given in SEM (Figure 1) even though exactly the same data were used. This is because in the PCA figures presented, the supplementary variable ‘Seed set 2013’ took up 1 degree of freedom to compose the PCA figures. This supplementary variable was not included in the PCAs that were used as data input for the SEM.

Overlay plot of carnivore community ordination in 2012 and 2013

The overlay plot of the carnivore community ordination in both years as presented in the main text, Figure 3a, is the overlay of Supplementary Figure 1a (carnivores 2012) and Supplementary Figure 1b (carnivores 2013). An overlay is used instead of simply composing a PCA in which both communities are included, because an ordination is determined by all species occurring on a plant (Šmilauer and Lepš, 2014). If the ordination would have been made on both communities in a single PCA-plot, as shown in Supplementary Figure 1c, the resulting ordination is different from the ordination of carnivores 2012 (Supplementary Figure 1b) and carnivores 2013 (Supplementary Figure 1b) separately.

In Supplementary Figure 1c (carnivores 2012 * carnivores 2013 in a single PCA), however, note the larger resemblance with the carnivore 2013-arrows of Supplementary Figure 1b (carnivores 2013), than is the case with the carnivore 2012-arrows of Supplementary Figure 1a (carnivores 2012). This is caused by the larger explained variation by PCA of the 2013-carnivore community composition compared to the 2012 carnivore community composition. (Compare % explained variation of first axis of Supplementary Figure 1a and 1b.)

References Supplementary material

Šmilauer, P., & Lepš, J. (2014). Multivariate analysis of ecological data using CANOCO 5. Cambridge university press.

Supplementary Table

Supplementary Table 1. Species encountered on the plants during both seasons, 2012 and 2013. Their functional group (herbivore / predator / parasitoid) is included; here, we grouped predators and parasitoids together as carnivores. [a] Species was only encountered during 2013 field season; [b] Only cocoons of the species were recorded; [c] Only mummified aphids were recorded.

Species	Order	Family	Functional group	Remark
Phyllotreta undulata	Coleoptera	Chrysomelidae	Herbivore	
Phyllotreta atra	Coleoptera	Chrysomelidae	Herbivore	
Coccinella spp.	Coleoptera	Coccinellidae	Predator	
Meligethes aeneus	Coleoptera	Nitidulidae	Herbivore	
Ceutorhynchus assimilis	Coleoptera	Curculionidae	Herbivore	
Cantharis spp.	Coleoptera	Cantharidae	Predator	2013 only[a]
Amphimallon solstitiale	Coleoptera	Scarabaeidae	Herbivore	2013 only
Unknown species of tortoise beetle	Coleoptera	Chrysomelidae	Herbivore	
Pieris rapae	Lepidoptera	Pieridae	Herbivore	
Pieris brassicae	Lepidoptera	Pieridae	Herbivore	
Plutella xylostella	Lepidoptera	Yponomeutidae	Herbivore	
Mamestra brassicae	Lepidoptera	Noctuidae	Herbivore	
Lacanobia suasa	Lepidoptera	Noctuidae	Herbivore	
Autographa gamma	Lepidoptera	Noctuidae	Herbivore	
Evergestis forficalis	Lepidoptera	Crambidae	Herbivore	
Several other species of *Lepidoptera*	Lepidoptera	-	Herbivore	2013 only
Brevicoryne brassicae	Hemiptera	Aphididae	Herbivore	

Myzus persicae	Hemiptera	Aphididae	Herbivore	
Other aphids than *B. brassicae* or *M. persicae*	Hemiptera	Aphididae	Herbivore	
Philaenus spp.	Hemiptera	Aphrophoridae	Herbivore	2013 only
Orius insidiosus	Hemiptera	Anthocoridae	Predator	
Lygus spp.	Hemiptera	Miridae	Herbivore	
Eurydema oleracea	Hemiptera	Pentatomidae	Herbivore	
Aleyrodes spp.	Hemiptera	Aleyrodidae	Herbivore	
Several species of *Symphyta* (sawfly) larvae	Hymenoptera	-	Herbivore	
Cotesia rubecula	Hymenoptera	Braconidae	Parasitoid	Cocoons[b]
Cotesia glomerata	Hymenoptera	Braconidae	Parasitoid	Cocoons
Microplitis mediator	Hymenoptera	Braconidae	Parasitoid	Cocoons
Praon spp.	Hymenoptera	Braconidae	Parasitoid	Cocoons
Species parasitizing *P. xylostella* (likely *Diadegma* spp.)	Hymenoptera	Ichneumonidae	Parasitoid	Cocoons
Several species parasitizing *Brevicoryne brassicae*	Hymenoptera	-	Parasitoid	Mummies[c]
Several species parasitizing *M. persicae*	Hymenoptera	-	Parasitoid	Mummies[c]
Several species parasitizing other aphids than *B. brassicae* or *M. persicae*	Hymenoptera	-	Parasitoid	Mummies[c]
Several species of Syrphidae larvae	Diptera	Syrphidae	Predator	
Unknown species of gall midge larvae	Diptera	Cecidomyiidae	Predator	
Unknown species of thrips	Thysanoptera	-	Herbivore	
Several species of Neuroptera	Neuroptera	-	Predator	
Several species of Spiders	Araneae	-	Predator	

Several species of snails and slugs	-	-	Herbivore	Class Gastropoda
Several (unknown) species of leaf mining insects	-	-	Herbivore	2013 only

Supplementary Figure

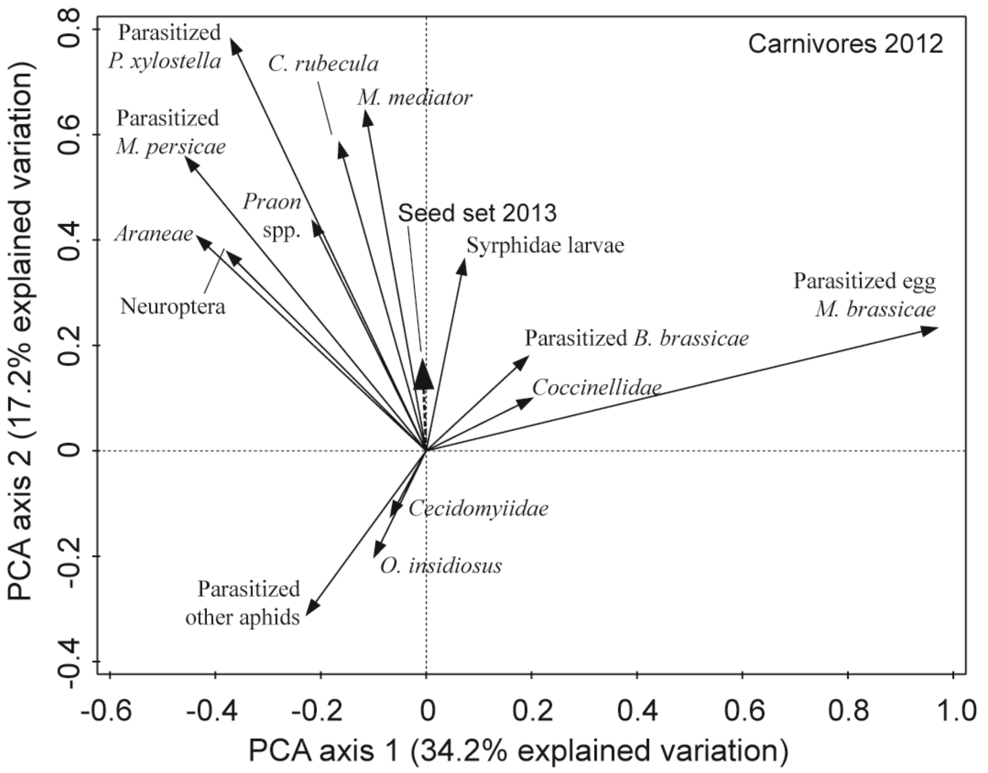

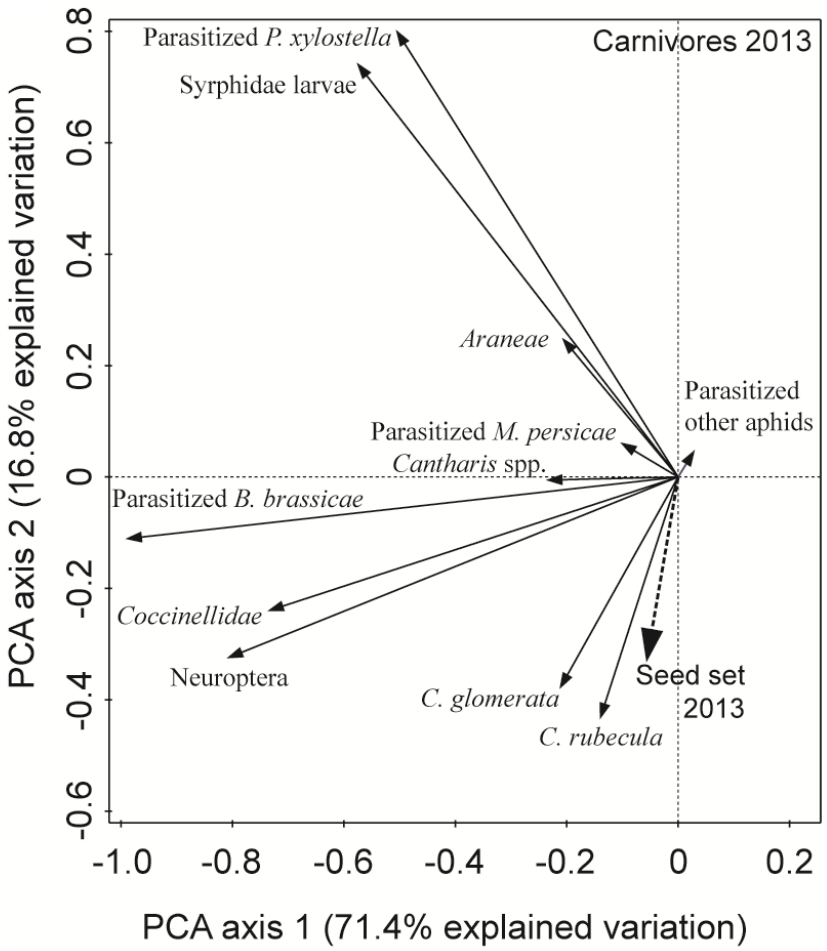
Carnivores 2013
Parasitized P. xylostella
Syrphidae larvae
Araneae
Parasitized other aphids
Parasitized M. persicae
Cantharis spp.
Parasitized B. brassicae
Coccinellidae
Neuroptera
C. glomerata
C. rubecula
Seed set 2013
PCA axis 1 (71.4% explained variation)
PCA axis 2 (16.8% explained variation)
-1.0
-0.8
-0.6
-0.4
-0.2
0
0.2
0.8
0.6
0.4
0.2
-0.2
-0.4
-0.6

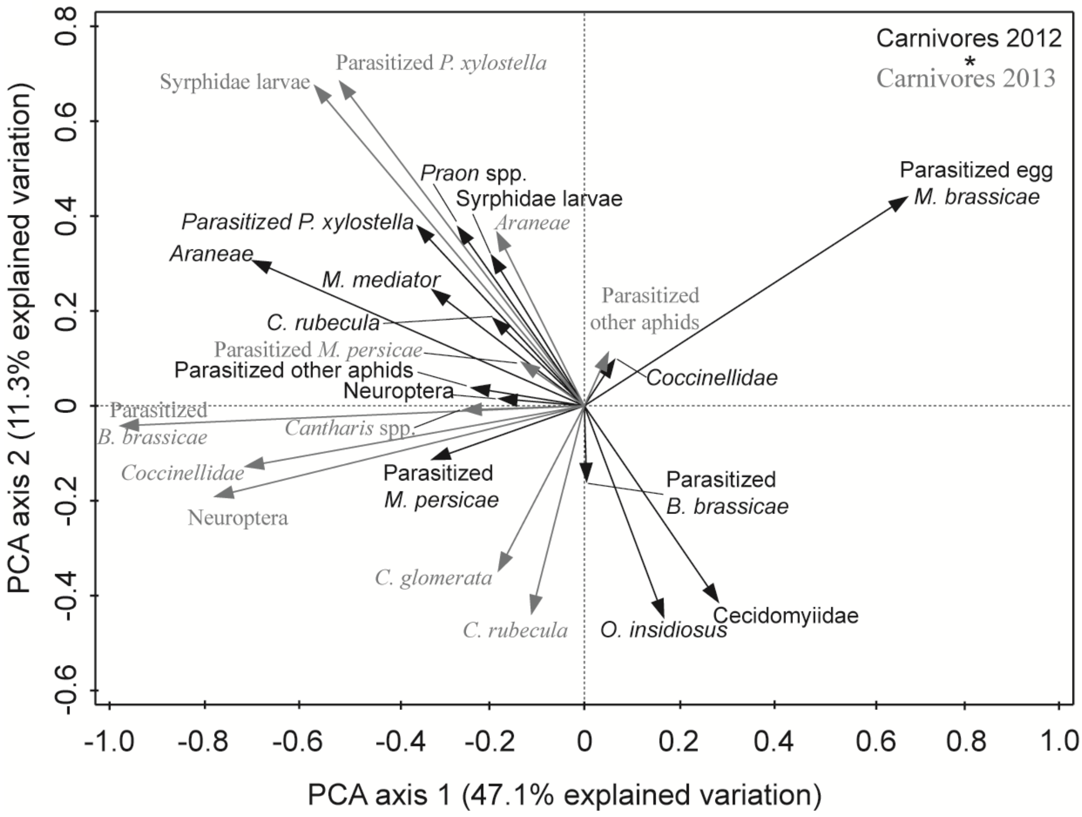

Supplementary Figure 1. Principal Component Analysis Ordination biplots of a) Carnivores 2012 with seed set 2013, b) Carnivores 2013 with seed set 2013 and c) Carnivores 2012 (black arrows) * Carnivores 2013 (grey arrows). Seed set 2013 was not included in the analyses of ordination, but projected on the plots afterwards. % explained variation in community composition by seed set is for a) 3.4% and b) 2.3%.
